# Supplementary material for: Divergent Morphologies and Common Signaling Features of Active and Inactive Oncogenic RHOA Mutants in Yeast
Source: Cells. 2025 Sep 15;14(18):1439. doi: 10.3390/cells14181439 (PMC12468435; doi:10.3390/cells14181439)
Supplement: Supplementary file 1 [file cells-14-01439-s001.zip › cells-3854339-supplementary.pdf]

Supplementary Materials

List of Figures:

Supplementary Figure S1. Growth phenotypes of *RHOA* variants in the presence of low-dose caspofungin.

Supplementary Figure S2. Quantification of Rho1 protein levels in *RHO1*/*RHO1* and *RHO1*/*RHO1 ade3* strains.

Supplementary Figure S3. Phosphorylation levels of Slt2 MAPK in the presence of *RHO1*.

Supplementary Figure S4. Cumulative contribution ratio of the first eight PCs calculated by PCA on mean Z-values of 501 morphological parameters.

Supplementary Figure S5. Cumulative contribution ratio of 100 PCs derived from 114 biological replicates of the standard strain BY4743.

Supplementary Figure S6. Morphological traits significantly altered in heterozygous *RHOA* mutants.

Supplementary Figure S7. Cumulative contribution ratio of the top 24 principal components on five strains.

List of Tables:

Supplementary Table S1. Yeast Strains Used in This Study.

Supplementary Table S2. Primers Used in This Study.

Supplementary Table S3. List of the 164 Parameters Detected by Likelihood Ratio Test for One-Way ANOVA Model at FDR < 0.05.

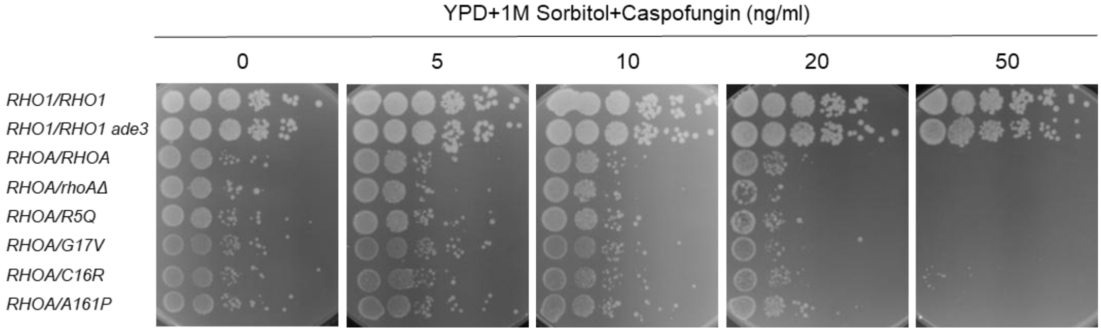

Supplementary Figure S1. Growth phenotypes of *RHOA* variants in the presence of low-dose caspofungin. Spotting assays on YPD + 1 M sorbitol with 5–50 ng/mL caspofungin showed minimal differences in colony growth across strains. Images were taken after 2 days of incubation.

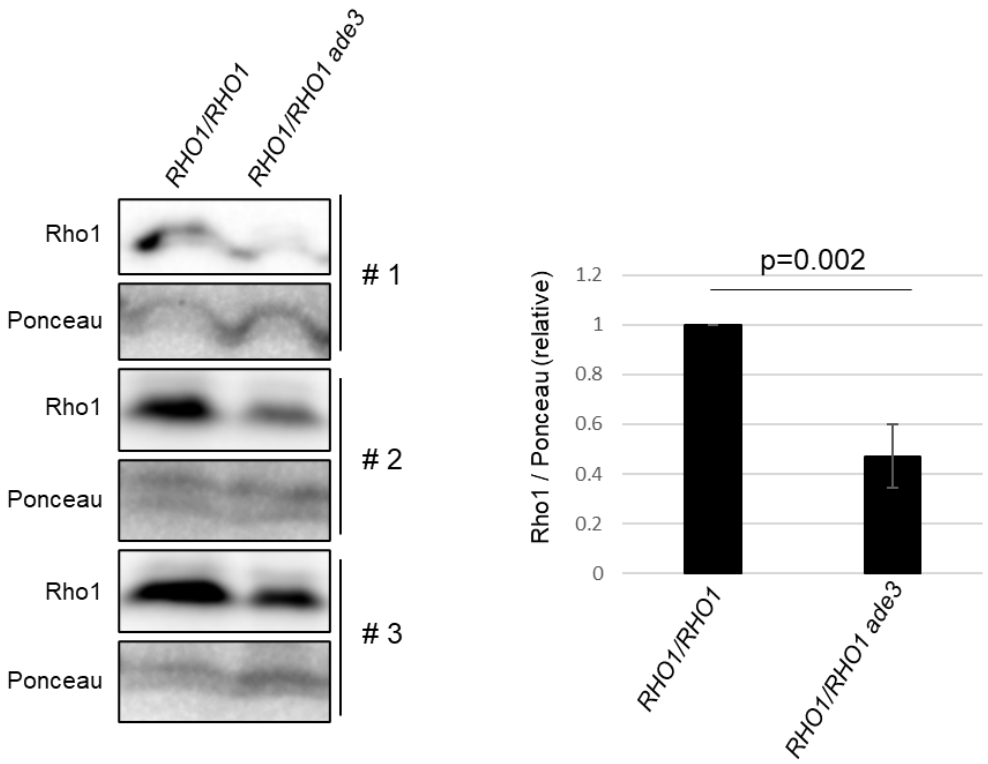

Supplementary Figure S2. Quantification of Rho1 protein levels in *RHO1*/*RHO1* and *RHO1*/*RHO1 ade3* strains. Western blotting using anti-Rho1 antibody revealed reduced protein expression in *RHO1*/*RHO1 ade3*, potentially explaining enhanced caspofungin sensitivity.

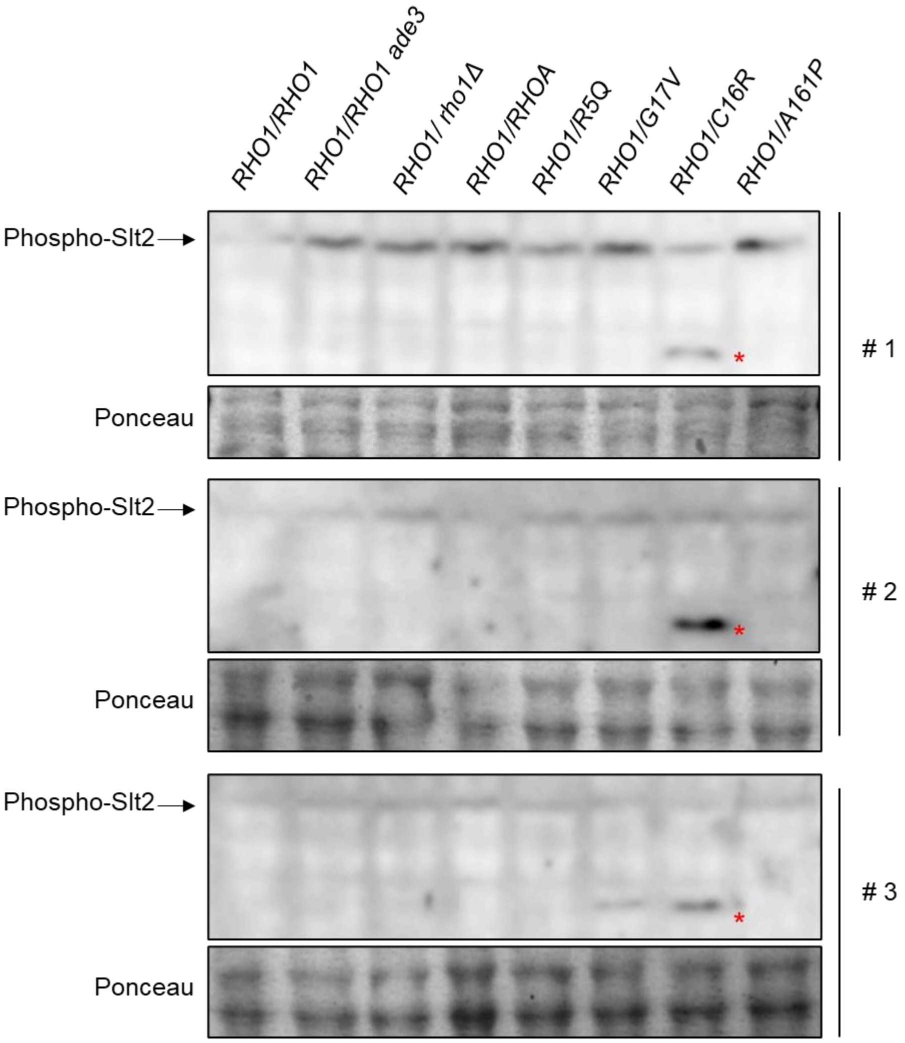

Supplementary Figure S3. Phosphorylation levels of Slt2 MAPK in the presence of *RHO1*. *RHOA* variants had no significant effect on Slt2 phosphorylation in the *RHO1*/*rho1Δ* background. The smaller phospho-band marked with a red star was consistently observed in *RHOA*/*C16R*, suggesting a potential novel posttranslational modification or degradation product.

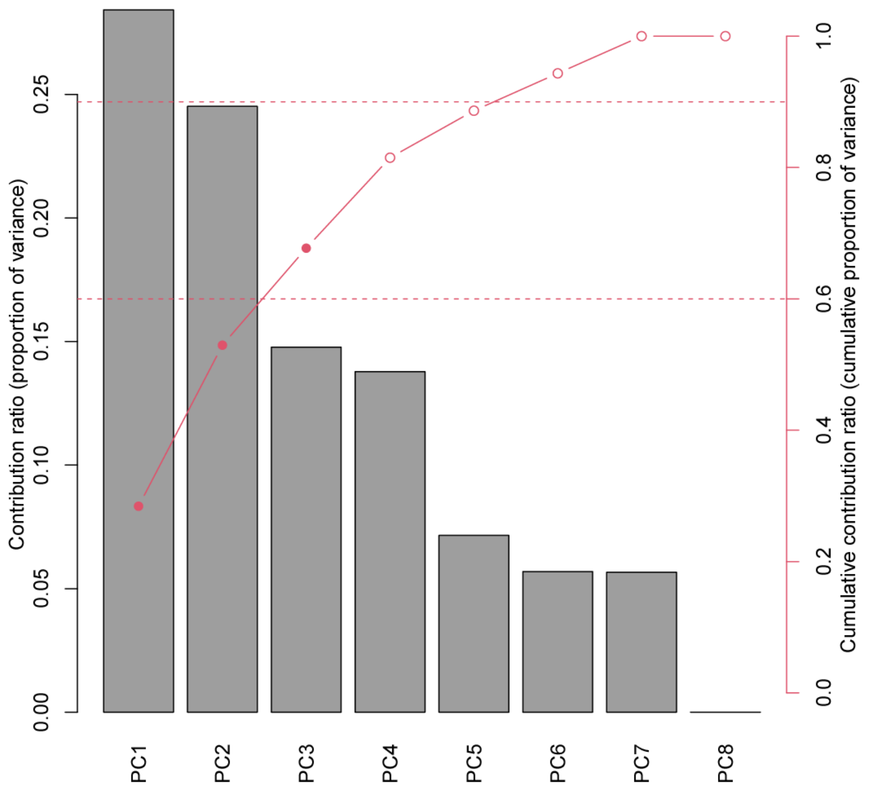

Supplementary Figure S4. Cumulative contribution ratio of the first eight PCs calculated by PCA on mean Z-values of 501 morphological parameters. Bars indicate the proportion of variance explained by each PC, and the red line represents the cumulative proportion of variance explained. Over 90% of the total variance was captured by the first six PCs, and the top three alone explained 60%.

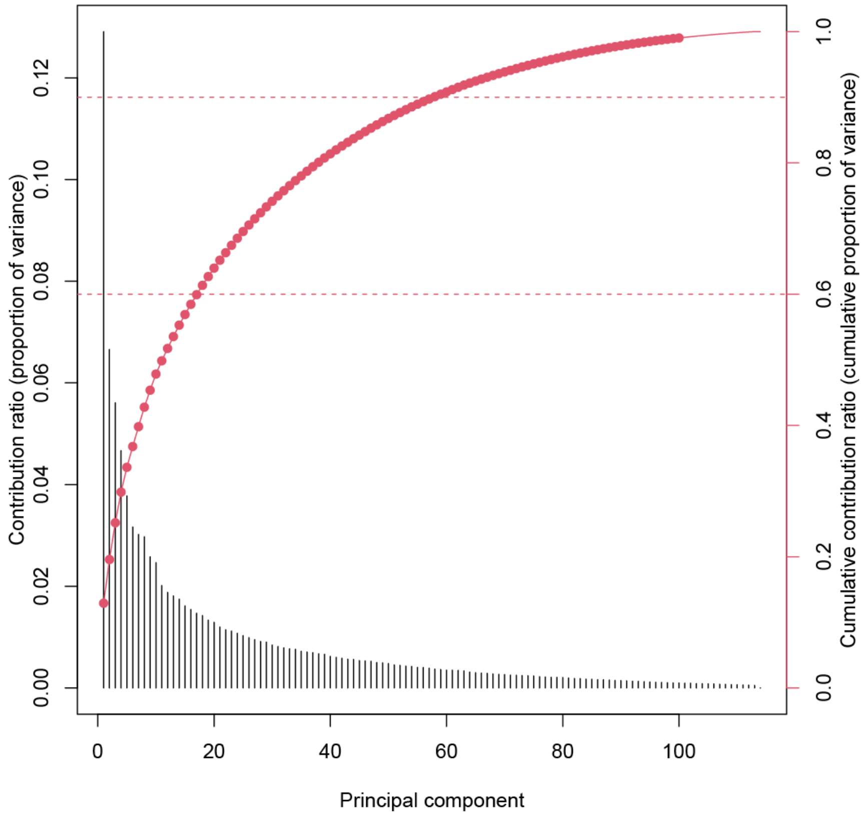

Supplementary Figure S5. Cumulative contribution ratio of 100 PCs derived from 114 biological replicates of the standard strain BY4743. More than 99% of the total variance was captured.

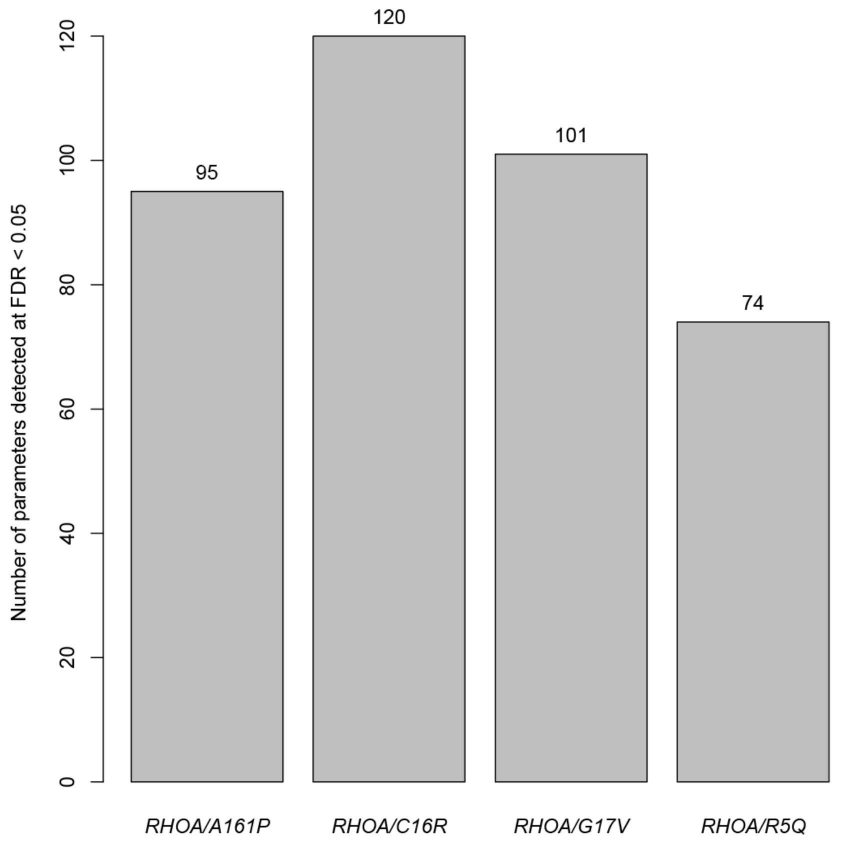

Supplementary Figure S6. Morphological traits significantly altered in heterozygous *RHOA* mutants. A total of 164 morphological parameters were found to be significantly fitted to the one-way ANOVA model across five *RHOA* strains (one control strain of *RHOA/rhoAΔ* and the four *RHOA* heterozygous mutant strains) by a generalized linear model with Storey's FDR correction (FDR < 0.05; likelihood ratio test). Bars and figures over the bars indicate the number of parameters detected at FDR < 0.05 by Wald test among the 156 parameters to have significant changes for each mutant from the control, with *RHOA/C16R* showing the most extensive phenotypic alterations.

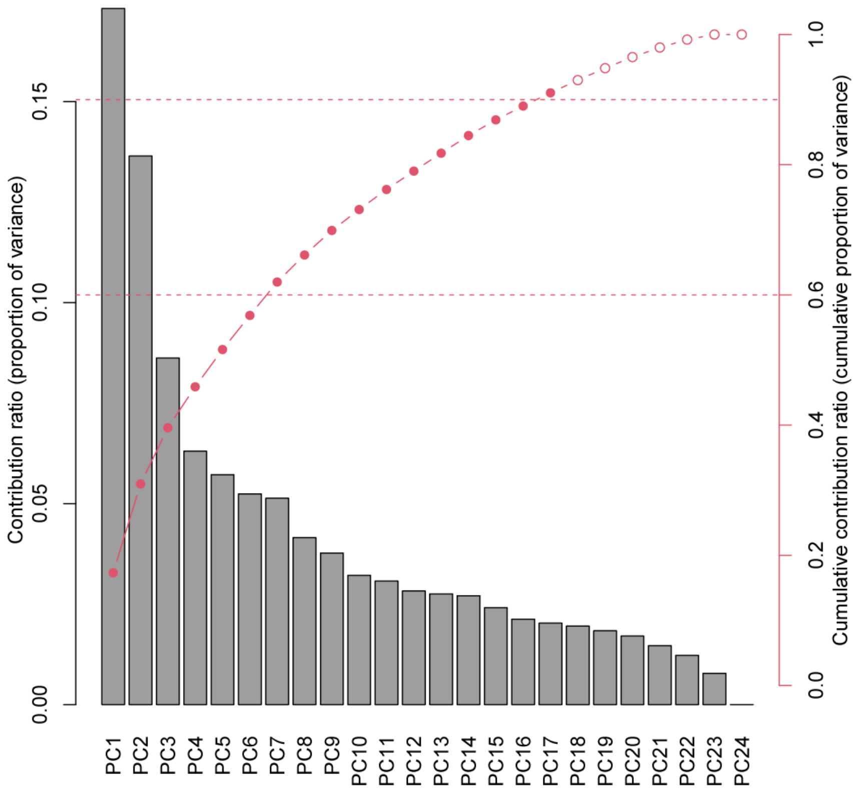

Supplementary Figure S7. Cumulative contribution ratio of the top 24 principal components on five strains. Bars indicate the variance explained by each PC, and the red line indicates the cumulative proportion. More than 90% of the variance was captured by the first 17 PCs.

Supplementary Table S1. Yeast Strains Used in This Study.

Alias (YOC number)	Mating type	Parental strains	Relevant genotype
RHO1 / RHO1 (YOC2260)	*MATa / MATα*	-	*ade2/ade2 his3/his3 leu2/leu2 lys2/lys2 trp1/trp1 ura3/ura3*
RHO1 α (ade3) (YOC764)	*MATα*	-	*ade2 his3 leu2 lys2 trp1 ura3 rho1::HIS3 ade3::[pRHO1-RHO1:LEU2]*
RHO1 a *(ade3)* (YOC784)	*MATa*	-	*ade2 his3 leu2 lys2 trp1 ura3 rho1::HIS3 ade3::[pRHO1-RHO1:LEU2]*
RHO1 / RHO1 (ade3) (YOC3410)	*MATa / MATα*	YOC764×YOC784	*ade2/ade2 his3/his3 leu2/leu2 lys2/lys2 trp1/trp1 ura3/ura3 rho1::HIS3/rho1::HIS3 ade3::[pRHO1-RHO1:LEU2]/ade3::[pRHO1-RHO1:LEU2]*
RHOA α (YOC763)	*MATα*	-	*ade2 his3 leu2 lys2 trp1 ura3 rho1::HIS3 ade3::[pRHO1-rhoA:LEU2]*
RHOA a (YOC783)	*MATa*	-	*ade2 his3 leu2 lys2 trp1 ura3 rho1::HIS3 ade3::[pRHO1-rhoA:LEU2]*
RHOA / RHOA (YOC3411)	*MATa / MATα*	YOC763×YOC783	*ade2/ade2 his3/his3 leu2/leu2 lys2/lys2 trp1/trp1 ura3/ura3 rho1::HIS3/rho1::HIS3 ade3::[pRHO1-rhoA:LEU2]/ade3::[pRHO1-rhoA:LEU2]*
YQ1512 (YOC708)	*MATα*	-	*ade2 his3 leu2 lys2 trp1 ura3 rho1::LYS2 [YEpU-RHO1]*
RHOA a (YOC783)	*MATa*	-	*ade2 his3 leu2 lys2 trp1 ura3 rho1::HIS3 ade3::[pRHO1-rhoA:LEU2]*
RHOA / rhoAΔ (YOC3412)	*MATa / MATα*	YOC708×YOC783	*ade2/ade2 his3/his3 leu2/leu2 lys2/lys2 trp1/trp1 ura3/ura3 rho1::LYS2/rho1::HIS3 ADE3/ade3::[pRHO1-rhoA:LEU2]*
R5Q	*MATα*	-	*ade2 his3 leu2 lys2 trp1 ura3 rho1::LYS2 ade3::rhoA-R5Q:LEU2 [YEpU-RHO1]*
G17V	*MATα*	-	*ade2 his3 leu2 lys2 trp1 ura3 rho1::LYS2 ade3::rhoA-G17V:LEU2 [YEpU-RHO1]*
C16R	*MATα*	-	*ade2 his3 leu2 lys2 trp1 ura3 rho1::LYS2 ade3::rhoA-C16R:LEU2 [YEpU-RHO1]*
A161P	*MATα*	-	*ade2 his3 leu2 lys2 trp1 ura3 rho1::LYS2 ade3::rhoA-A161P:LEU2 [YEpU-RHO1]*
E40Q	*MATα*	-	*ade2 his3 leu2 lys2 trp1 ura3 rho1::LYS2 ade3::rhoA-E40Q:LEU2 [YEpU-RHO1]*

RHOA / R5Q (YOC3413)	*MATa / MATα*	*R5Q*×YOC783	*ade2/ade2 his3/his3 leu2/leu2 lys2/lys2 trp1/trp1 ura3/ura3 rho1::HIS3/rho1::LYS2 ade3::[pRHO1-rhoA:LEU2]/ade3::rhoA-R5Q:LEU2*
RHOA / G17V (YOC3414)	*MATa / MATα*	*G17V*×YOC783	*ade2/ade2 his3/his3 leu2/leu2 lys2/lys2 trp1/trp1 ura3/ura3 rho1::HIS3/rho1::LYS2 ade3::[pRHO1-rhoA:LEU2]/ade3::rhoA-G17V:LEU2*
RHOA / C16R (YOC3415)	*MATa / MATα*	*C16R*×YOC783	*ade2/ade2 his3/his3 leu2/leu2 lys2/lys2 trp1/trp1 ura3/ura3 rho1::HIS3/rho1::LYS2 ade3::[pRHO1-rhoA:LEU2]/ade3::rhoA-C16R:LEU2*
RHOA / A161P (YOC3416)	*MATa / MATα*	*A161P*×YOC783	*ade2/ade2 his3/his3 leu2/leu2 lys2/lys2 trp1/trp1 ura3/ura3 rho1::HIS3/rho1::LYS2 ade3::[pRHO1-rhoA:LEU2]/ade3::rhoA-A161P:LEU2*
RHO1 / R5Q (YOC3417)	*MATa / MATα*	*R5Q*×YOC784	*ade2/ade2 his3/his3 leu2/leu2 lys2/lys2 trp1/trp1 ura3/ura3 rho1::HIS3/rho1::LYS2 ade3::[pRHO1-RHO1:LEU2]/ade3::rhoA-R5Q:LEU2*
RHO1 / G17V (YOC3418)	*MATa / MATα*	*G17V*×YOC784	*ade2/ade2 his3/his3 leu2/leu2 lys2/lys2 trp1/trp1 ura3/ura3 rho1::HIS3/rho1::LYS2 ade3::[pRHO1-RHO1:LEU2]/ade3::rhoA-G17V:LEU2*
RHO1 / C16R (YOC3419)	*MATa / MATα*	*C16R*×YOC784	*ade2/ade2 his3/his3 leu2/leu2 lys2/lys2 trp1/trp1 ura3/ura3 rho1::HIS3/rho1::LYS2 ade3::[pRHO1-RHO1:LEU2]/ade3::rhoA-C16R:LEU2*
RHO1 / A161P (YOC3420)	*MATa / MATα*	*A161P*×YOC784	*ade2/ade2 his3/his3 leu2/leu2 lys2/lys2 trp1/trp1 ura3/ura3 rho1::HIS3/rho1::LYS2 ade3::[pRHO1-RHO1:LEU2]/ade3::rhoA-A161P:LEU2*
YEpU-*RHO1* (YOC5638)	*MATα*	-	*ade2 his3 leu2 lys2 trp1 ura3 rho1::LYS2 ade3::LEU2 [YEpU-RHO1]*
RHO1 / rho1Δ	*MATa / MATα*	YOC5638×YOC784	*ade2/ade2 his3/his3 leu2/leu2 lys2/lys2 trp1/trp1 ura3/ura3 rho1::HIS3/rho1::LYS2 ade3::LEU2/ade3::[pRHO1-RHO1:LEU2]*
RHO1 / RHOA	*MATa / MATα*	YOC763×YOC784	*ade2/ade2 his3/his3 leu2/leu2 lys2/lys2 trp1/trp1 ura3/ura3 rho1::HIS3/rho1::HIS3 ade3::[pRHO1-RHO1:LEU2]/ade3::[pRHO1-rhoA:LEU2]*

Supplementary Table S2. Primers Used in This Study.

Name	Forward (5′→3′)	Reverse (5′→3′)
p-pKN4	ACTTGTTGCCAAAGAAGTCTGCCAAGACTGCCAAAGCTTCTCAAGAACTGcggatccccgggttaattaac	CAAATGACAAGAATGTTTGATTTGCTTTGTTTCTTTTCAACTCAGTTCTTAgaattcgagctcgtttaaac
p-pKN23	CTTCGAAGAATATACTAAAAAATGAGCAGGCAAGATAAACGAAGGCAAAGacgcggccgccagctgaagc	CACATGTATATATATCGTATGCTGCAGCTTTAAATAATCGGTGTCACTACcagtatagcgaccagcattc

Supplementary Table S3. List of the 164 Parameters Detected by Likelihood Ratio Test for One-Way ANOVA Model at FDR < 0.05.

		Z value					Wald test (FDR)				
ID	LRT (FDR)	*RHOA/rhoAΔ*	*RHOA/A161P*	*RHOA/C16R*	*RHOA/G17V*	*RHOA/R5Q*	*RHOA/A161P*	*RHOA/C16R*	*RHOA/G17V*	*RHOA/R5Q*	Description
D209	0.0000		[illegible]	[illegible]	-1.090	-0.502	0.2685	0.2581	0.1066	0.1947	Nuclear_C_ratio_to_budded_cells
A118	0.0001		-4.923	-5.103	-6.531	-1.310	0.0006	0.0005	0.0002	0.0827	Actin_e_ratio_to_budded_cells
A9_A1B	0.0001		4.198	6.305	6.040	4.495	0.0014	0.0002	0.0002	0.0010	Proportion_of_actin_region_at_neck
A109_A1B	0.0004		-3.322	-4.037	-5.900	0.249	0.0049	0.0017	0.0002	0.2356	Actin_e_ratio
C103_A	0.0007		-5.548	-5.493	-3.868	-5.363	0.0003	0.0003	0.0022	0.0003	Long_axis_length_in_whole_cell
DCV196_A1B	0.0007		2.628	3.664	6.175	-0.360	0.0141	0.0037	0.0002	0.2183	Noise_of_maximal_intensity_of_nuclear_brightness_divided_by_average
C126_C	0.0007		0.890	-4.139	-0.758	-1.173	0.1342	0.0020	0.1540	0.0986	Brightness_difference_of_cell_wall
DCV15-1_C	0.0007		3.188	9.891	2.952	1.402	0.0068	0.0000	0.0092	0.0756	Noise_of_nuclear_brightness_in_mother
C116_A1B	0.0008		5.712	3.723	5.127	2.076	0.0002	0.0027	0.0005	0.0288	Axis_ratio_ratio
ACV8-1_A1B	0.0013		4.658	2.890	4.054	[illegible]	0.0008	0.0086	0.0017	0.2671	Noise_of_total_brightness_of_actin_region_in_mother
A102_A1B	0.0020		1.772	0.435	2.161	-3.135	0.0442	0.2052	0.0254	0.0062	Bud_actin_region_ratio_to_total_region
CCV115_A1B	0.0024		2.194	4.282	6.766	3.145	0.0258	0.0017	0.0002	0.0069	Noise_of_mother_axis_ratio
DCV158_C	0.0024		2.147	[illegible]	-3.213	0.985	0.0263	0.2257	0.0057	0.1209	Noise_of_angle_between_D1-1D1-2_and_C1-1C1-2
C126_A	0.0030		0.630	-5.110	-0.898	-1.342	0.1737	0.0007	0.1333	0.0812	Brightness_difference_of_cell_wall
C126_A1B	0.0030		0.160	-6.030	-1.891	-2.152	0.2496	0.0003	0.0386	0.0273	Brightness_difference_of_cell_wall
DCV159_C	0.0030		1.517	-0.830	-3.567	0.477	0.0644	0.1418	0.0036	0.1995	Noise_of_angle_between_D2-1D2-2_and_C1-1C1-2
A109	0.0030		-2.433	-3.941	-4.674	-1.217	0.0166	0.0019	0.0007	0.0922	Actin_e_ratio
D15-3_A1B	0.0031		4.248	6.534	5.343	1.493	0.0017	0.0002	0.0005	0.0679	Nuclear_brightness
A123_A	0.0045		-0.858	-4.366	-0.369	-1.840	0.1389	0.0015	0.2171	0.0416	Ratio_of_actin_patches_to_actin_region
D172_A1B	0.0045		2.063	6.391	3.138	1.331	0.0308	0.0002	0.0069	0.0821	Angle_between_C4-1D4_and_C4-1C1
D170_C	0.0045		1.683	3.777	3.219	1.008	0.0522	0.0032	0.0063	0.1183	Angle_between_C4-1D2-1_and_C4-1C1
DCV17-1_C	0.0045		1.950	5.309	[illegible]	1.156	0.0363	0.0007	0.2695	0.1008	Noise_of_nuclear_fitness_for_ellipse_in_mother
D215	0.0057		3.219	[illegible]	1.319	-0.390	0.0054	0.2367	0.0819	0.2132	Nuclear_B_ratio_to_nuclear_A1BC_cells
A101_A	0.0060		1.730	4.257	1.361	2.037	0.0488	0.0017	0.0792	0.0319	Actin_region_ratio_in_whole_cell
ACV122_A1B	0.0060		2.814	4.845	3.127	1.378	0.0095	0.0007	0.0062	0.0757	Noise_of_number_of_bright_actin_patches
D169_C	0.0060		1.716	5.400	2.446	0.471	0.0496	0.0005	0.0180	0.2005	Angle_between_C4-1D1-1_and_C4-1C1
D208	0.0060		2.545	-0.464	0.166	-1.384	0.0142	0.2012	0.2491	0.0754	Nuclear_B_ratio_to_budded_cells
D201	0.0062		2.649	-0.460	0.279	-1.185	0.0121	0.2016	0.2308	0.0955	Nuclear_B_ratio
DCV191_C	0.0076		2.284	4.728	1.579	1.239	0.0218	0.0008	0.0590	0.0904	Noise_of_average_of_nuclear_brightness_in_mother
D212	0.0076		2.993	[illegible]	1.154	-0.605	0.0071	0.2355	0.0993	0.1765	Nuclear_B_ratio_to_nuclear_AA1BC_cells
D15-1_A	0.0076		2.920	4.010	4.834	2.269	0.0092	0.0023	0.0009	0.0235	Nuclear_brightness
CCV103_A1B	0.0076		1.172	4.648	2.325	1.160	0.0974	0.0008	0.0198	0.0988	Noise_of_long_axis_length_in_mother
CCV128_A1B	0.0076		[illegible]	4.123	1.955	0.528	0.2698	0.0016	0.0340	0.1906	Noise_of_distance_between_middle_point_of_neck_and_mother_hip
A109_C	0.0076		-2.881	-3.793	-4.002	-1.474	0.0087	0.0025	0.0018	0.0680	Actin_e_ratio
A121_C	0.0076		-4.080	-4.126	-3.340	-2.141	0.0017	0.0016	0.0048	0.0263	Maximal_distance_between_patches
A117	0.0076		3.594	3.832	3.211	2.627	0.0033	0.0023	0.0056	0.0126	Actin_d_iso_ratio_to_budded_cells
A123_C	0.0076		-2.623	-4.371	-2.399	-3.619	0.0126	0.0012	0.0179	0.0032	Ratio_of_actin_patches_to_actin_region
C115_A1B	0.0083		3.334	4.328	4.057	3.088	0.0048	0.0012	0.0017	0.0066	Mother_axis_ratio
A101_C	0.0084		2.841	4.070	2.173	3.788	0.0092	0.0017	0.0254	0.0025	Actin_region_ratio_in_whole_cell
DCV15-3_C	0.0084		0.691	4.344	[illegible]	0.960	0.1636	0.0017	0.2746	0.1250	Noise_of_nuclear_brightness_in_whole_cell
DCV190_A1B	0.0084		2.256	4.422	3.766	2.102	0.0224	0.0011	0.0025	0.0276	Noise_of_distance_between_nuclear_gravity_center_and_brightest_point
A116	0.0085		1.455	1.433	2.851	-1.532	0.0696	0.0716	0.0091	0.0628	Actin_c_api_ratio_to_budded_cells
DCV169_A1B	0.0091		3.701	0.559	1.045	-0.744	0.0028	0.1851	0.1121	0.1556	Noise_of_angle_between_C4-1D1-1_and_C4-1C1
DCV139_C	0.0094		-1.412	-2.828	-5.547	-1.124	0.0751	0.0110	0.0005	0.1042	Noise_of_distance_between_nuclear_brightest_point_in_bud_and_bud_tip
D147_A	0.0100		1.995	4.081	2.955	1.734	0.0338	0.0021	0.0089	0.0487	Relative_distance_of_nuclear_gravity_center_to_cell_center
A112_A1B	0.0100		2.745	2.068	2.348	-1.095	0.0106	0.0290	0.0193	0.1063	Actin_cd_ratio
DCV147_A1B	0.0100		1.758	4.919	2.983	2.696	0.0468	0.0008	0.0085	0.0126	Noise_of_relative_distance_of_nuclear_gravity_center_to_mother_center
C115_C	0.0100		2.906	4.248	3.647	2.213	0.0084	0.0014	0.0030	0.0238	Mother_axis_ratio
DCV121_C	0.0100		-0.836	-3.063	-5.134	-0.506	0.1418	0.0079	0.0007	0.1947	Noise_of_distance_between_nuclear_gravity_center_in_bud_and_bud_tip
A105	0.0100		1.974	3.218	0.877	2.274	0.0331	0.0054	0.1348	0.0215	Actin_a_ratio
A8-1_A	0.0101		3.520	4.596	2.805	3.460	0.0037	0.0008	0.0096	0.0039	Actin_region_brightness
CCV13_C	0.0101		4.912	2.306	1.444	5.246	0.0009	0.0227	0.0720	0.0007	Noise_of_mother_cell_fitness_for_ellipse
A112_C	0.0102		3.347	3.320	3.499	2.109	0.0048	0.0049	0.0037	0.0274	Actin_cd_ratio
CCV117_C	0.0102		2.970	5.206	1.072	2.529	0.0091	0.0007	0.1102	0.0164	Noise_of_cell_outline_ratio
A113_A	0.0145		[illegible]	1.607	1.989	-1.638	0.2455	0.0562	0.0326	0.0542	Actin_n_ratio
D198_C	0.0146		2.167	2.552	1.640	-0.444	0.0266	0.0158	0.0553	0.2042	Ratio_of_nuclear_brightness
C128_A1B	0.0149		-5.925	-3.607	-6.591	-6.694	0.0003	0.0039	0.0002	0.0002	Distance_between_middle_point_of_neck_and_mother_hip
A108	0.0149		3.494	3.332	2.397	2.435	0.0038	0.0048	0.0179	0.0167	Actin_d_iso_ratio
C103_A1B	0.0170		-6.189	-3.808	-6.636	-6.699	0.0002	0.0030	0.0002	0.0002	Long_axis_length_in_mother
A8-2_C	0.0170		4.150	3.856	2.841	3.230	0.0015	0.0022	0.0092	0.0054	Total_brightness_of_actin_region_in_bud
C112_A1B	0.0179		-5.358	-3.039	-5.738	-5.809	0.0005	0.0079	0.0003	0.0003	Distance_between_middle_point_of_neck_and_mother_center
A120_C	0.0184		-3.432	-3.413	-3.122	-1.701	0.0041	0.0042	0.0062	0.0491	Total_length_of_actin_patch_link
ACV121_A	0.0192		-2.710	-3.130	-2.339	-4.068	0.0112	0.0062	0.0195	0.0017	Noise_of_maximal_distance_between_patches
C115_A	0.0195		2.926	3.529	3.682	3.016	0.0081	0.0037	0.0029	0.0071	Whole_cell_axis_ratio
D130_C	0.0195		-1.299	-4.447	-2.882	-0.761	0.0850	0.0014	0.0096	0.1540	Distance_between_nuclear_brightest_point_in_mother_and_middle_point_of_neck
D143_C	0.0195		-1.135	-2.978	-3.517	-0.731	0.1014	0.0075	0.0037	0.1566	Distance_between_nuclear_outline_point_D6-1_in_mother_and_middle_point_of_neck
A107	0.0195		1.406	0.814	1.737	-1.955	0.0737	0.1440	0.0467	0.0340	Actin_c_api_ratio
C127_A	0.0195		-1.472	-2.262	1.308	-1.913	0.0680	0.0221	0.0827	0.0361	Thickness_difference_of_cell_wall
A7-1_A	0.0195		1.602	3.418	1.529	1.946	0.0584	0.0051	0.0644	0.0360	Size_of_actin_region
A105_A	0.0199		2.437	3.097	1.289	3.458	0.0167	0.0065	0.0849	0.0039	Actin_a_ratio
CCV13_A	0.0199		-1.946	0.914	0.614	-0.735	0.0360	0.1308	0.1758	0.1566	Noise_of_whole_cell_fitness_for_ellipse
A103_A1B	0.0199		-0.731	-1.476	-2.038	1.787	0.1566	0.0679	0.0303	0.0433	Relative_distance_of_actin_patch_center_from_neck_in_mother
D16-3_C	0.0199		4.154	3.058	4.549	1.936	0.0019	0.0077	0.0013	0.0364	Maximal_intensity_of_nuclear_brightness_in_whole_cell
DCV150_C	0.0199		-0.976	-1.779	[illegible]	-3.547	0.1229	0.0462	0.2263	0.0046	Noise_of_relative_distance_of_nuclear_brightest_point_in_bud_to_bud_center
DCV161_A1B	0.0209		0.495	2.261	0.616	-0.260	0.1962	0.0237	0.1756	0.2345	Noise_of_angle_between_D3-1D4-1_and_C1-1C1-2_or_between_D3-3D4-3_and_C1-1C1-2
D204	0.0209		[illegible]	[illegible]	1.117	0.835	0.2496	0.2556	0.1036	0.1411	Nuclear_E_ratio
A101_A1B	0.0230		1.556	3.176	0.944	3.245	0.0606	0.0058	0.1260	0.0054	Actin_region_ratio_in_whole_cell
D192_C	0.0230		4.651	4.449	5.119	1.640	0.0011	0.0014	0.0007	0.0553	Average_of_nuclear_brightness_in_bud
DCV16-3_C	0.0230		0.715	2.477	[illegible]	-1.233	0.1593	0.0162	0.2491	0.0908	Noise_of_maximal_intensity_of_nuclear_brightness_in_whole_cell
A7-2_A1B	0.0231		1.097	2.234	1.208	0.317	0.1068	0.0246	0.0943	0.2243	Size_of_actin_region_in_bud
D206	0.0231		0.159	-0.277	-0.789	0.151	0.2496	0.2311	0.1484	0.2505	Nuclear_A_ratio_to_no_bud_cells
CCV108_A1B	0.0234		0.330	2.772	3.248	1.617	0.2222	0.0101	0.0054	0.0554	Noise_of_short_axis_length_in_bud
A121_A	0.0237		-2.053	-2.406	-1.589	[illegible]	0.0311	0.0191	0.0594	0.2717	Maximal_distance_between_patches
A107_A1B	0.0237		1.899	1.232	1.879	-1.390	0.0367	0.0908	0.0378	0.0751	Actin_c_api_ratio
D152_C	0.0237		1.442	-0.927	[illegible]	1.352	0.0720	0.1290	0.2382	0.0803	Mobility_of_nucleus_in_mother
CCV112_A1B	0.0237		0.567	2.871	1.984	0.595	0.1843	0.0098	0.0340	0.1790	Noise_of_distance_between_middle_point_of_neck_and_mother_center
DCV194_A	0.0241		1.818	3.444	2.110	0.941	0.0430	0.0049	0.0288	0.1273	Noise_of_maximal_intensity_of_nuclear_brightness_divided_by_average
DCV170_A1B	0.0241		2.212	[illegible]	0.336	-1.779	0.0238	0.2599	0.2211	0.0438	Noise_of_angle_between_C4-1D2-1_and_C4-1C1
A108_C	0.0241		2.865	2.939	3.019	1.902	0.0089	0.0079	0.0071	0.0366	Actin_d_iso_ratio
DCV182_C	0.0241		[illegible]	2.469	-1.141	-0.238	0.2746	0.0163	0.1013	0.2367	Noise_of_nuclear_axis_ratio_in_mother
DCV196_C	0.0241		1.212	3.196	1.332	-0.244	0.0929	0.0058	0.0812	0.2361	Noise_of_maximal_intensity_of_nuclear_brightness_divided_by_average_in_whole_cell
C13_A	0.0255		-2.559	-3.141	-2.518	-3.437	0.0142	0.0061	0.0151	0.0041	Whole_cell_fitness_for_ellipse
CCV106_A1B	0.0255		0.891	1.904	1.239	4.755	0.1342	0.0380	0.0911	0.0010	Noise_of_bud_direction
CCV103_C	0.0255		0.472	3.329	0.258	1.039	0.2003	0.0050	0.2345	0.1132	Noise_of_long_axis_length_in_mother
CCV128_C	0.0255		0.352	2.743	-0.737	1.239	0.2191	0.0110	0.1566	0.0904	Noise_of_distance_between_middle_point_of_neck_and_mother_hip
D105_A	0.0261		1.573	4.492	3.873	0.870	0.0606	0.0014	0.0028	0.1370	Ratio_of_D102_to_C103
CCV103_A	0.0261		-2.786	-0.648	-1.818	-3.291	0.0099	0.1707	0.0414	0.0051	Noise_of_long_axis_length_in_whole_cell
C117_A1B	0.0261		1.542	-0.641	[illegible]	-0.758	0.0635	0.1720	0.2506	0.1540	Cell_outline_ratio
CCV12-1_A1B	0.0261		0.655	3.507	1.697	0.545	0.1694	0.0037	0.0493	0.1874	Noise_of_mother_cell_outline_length
C111_C	0.0261		-1.127	-1.449	-1.338	-4.099	0.1036	0.0716	0.0815	0.0021	Distance_between_bud_tip_and_mother_short_axis_extension
D106_C	0.0261		-0.836	0.407	1.511	-2.165	0.1411	0.2101	0.0644	0.0254	Ratio_of_D103_to_C103
CCV11-2_C	0.0261		0.248	0.646	-1.970	1.589	0.2356	0.1708	0.0338	0.0583	Noise_of_bud_cell_size
DCV193_C	0.0261		1.428	3.594	0.953	0.899	0.0720	0.0035	0.1250	0.1325	Noise_of_average_of_nuclear_brightness_in_whole_cell
A113	0.0261		0.352	1.408	1.917	0.916	0.2191	0.0751	0.0373	0.1307	Actin_n_ratio
ACV123_A1B	0.0261		1.973	3.203	3.263	1.253	0.0334	0.0056	0.0053	0.0889	Noise_of_ratio_of_actin_patches_to_actin_region
D148_A	0.0266		2.054	3.127	2.502	2.764	0.0311	0.0070	0.0166	0.0116	Relative_distance_of_nuclear_brightest_point_to_cell_center
DCV188_A	0.0276		1.952	2.166	3.725	2.675	0.0340	0.0254	0.0027	0.0118	Noise_of_distance_between_nuclear_gravity_center_and_brightest_point
D178_A1B	0.0276		1.379	0.954	2.575	-1.011	0.0757	0.1250	0.0138	0.1169	Nuclear_long_axis_length
D108_C	0.0276		-1.136	-2.457	-3.394	-0.735	0.1014	0.0163	0.0044	0.1566	Distance_between_nuclear_gravity_center_in_mother_and_middle_point_of_neck
D14-1_A	0.0277		0.450	0.755	3.265	2.135	0.2029	0.1540	0.0053	0.0264	Nuclear_size
C111_A1B	0.0282		-1.853	-2.902	-3.265	-2.495	0.0409	0.0094	0.0060	0.0167	Distance_between_bud_tip_and_mother_short_axis_extension
A8-2_A1B	0.0282		3.373	4.121	2.721	0.356	0.0054	0.0020	0.0122	0.2190	Total_brightness_of_actin_region_in_bud
D170_A1B	0.0284		2.479	2.629	3.549	3.048	0.0160	0.0126	0.0036	0.0068	Angle_between_C4-1D2-1_and_C4-1C1
ACV8-1_C	0.0284		2.251	2.145	1.650	-0.557	0.0245	0.0280	0.0553	0.1859	Noise_of_total_brightness_of_actin_region_in_mother
ACV7-1_C	0.0286		2.181	3.224	1.705	1.206	0.0266	0.0066	0.0510	0.0949	Noise_of_size_of_actin_region_in_mother
D197_C	0.0287		2.549	2.584	1.994	1.273	0.0158	0.0151	0.0338	0.0879	Ratio_of_nuclear_size
ACV122_A	0.0287		2.465	2.331	2.052	-0.291	0.0162	0.0197	0.0297	0.2287	Noise_of_number_of_bright_actin_patches
DCV165_A1B	0.0292		-0.324	1.290	0.194	-0.329	0.2230	0.0860	0.2452	0.2224	Noise_of_angle_between_D3-1D4-1_and_C1C4-1_or_between_D3-3D4-3_and_C1C4-1
A123_A1B	0.0300		[illegible]	-2.497	[illegible]	-2.364	0.2599	0.0156	0.2705	0.0187	Ratio_of_actin_patches_to_actin_region
D16-2_C	0.0300		4.010	3.179	3.900	1.465	0.0023	0.0066	0.0027	0.0700	Maximal_intensity_of_nuclear_brightness_in_bud
DCV16-1_C	0.0300		2.395	3.304	1.145	0.842	0.0181	0.0052	0.1008	0.1409	Noise_of_maximal_intensity_of_nuclear_brightness_in_mother
CCV101_A1B	0.0303		1.058	3.548	2.197	1.424	0.1103	0.0036	0.0244	0.0720	Noise_of_whole_cell_size
D14-3_A1B	0.0319		2.171	2.062	2.912	[illegible]	0.0254	0.0293	0.0083	0.2587	Nuclear_size
CCV102_A1B	0.0320		1.058	3.461	2.373	1.952	0.1103	0.0039	0.0185	0.0340	Noise_of_whole_cell_outline_length
DCV14-1_A	0.0332		0.736	2.613	2.579	2.517	0.1566	0.0144	0.0151	0.0163	Noise_of_nuclear_size
C12-1_A1B	0.0332		-3.182	-2.751	-2.036	-2.981	0.0058	0.0106	0.0303	0.0075	Mother_cell_outline_length
ACV8-2_C	0.0340		1.662	0.583	0.512	-0.339	0.0544	0.1813	0.1941	0.2211	Noise_of_total_brightness_of_actin_region_in_bud
C106_C	0.0353		-2.082	-1.093	-1.640	-0.379	0.0299	0.1072	0.0553	0.2154	Bud_direction
D191_C	0.0357		3.048	2.711	3.178	2.722	0.0068	0.0112	0.0058	0.0110	Average_of_nuclear_brightness_in_mother
A113_A1B	0.0368		[illegible]	0.926	1.789	[illegible]	0.2685	0.1282	0.0430	0.2505	Actin_n_ratio
ACV8-2_A1B	0.0368		2.212	2.485	2.692	3.756	0.0254	0.0169	0.0126	0.0033	Noise_of_total_brightness_of_actin_region_in_bud
C105_C	0.0368		1.679	2.985	1.427	2.537	0.0524	0.0085	0.0732	0.0160	Neck_position
DCV134_C	0.0368		2.056	-0.372	2.315	0.414	0.0299	0.2154	0.0206	0.2090	Noise_of_distance_between_two_nuclear_brightest_points_through_middle_point_of_neck
A111	0.0368		-1.063	-1.516	-4.278	0.438	0.1099	0.0640	0.0012	0.2048	Actin_ae_ratio
D193_C	0.0374		3.916	3.954	4.656	2.042	0.0026	0.0025	0.0011	0.0317	Average_of_nuclear_brightness_in_whole_cell
A7-1_C	0.0386		1.868	2.680	0.684	2.980	0.0383	0.0118	0.1638	0.0075	Size_of_actin_region_in_mother
CCV104_A	0.0399		1.505	2.311	1.959	-0.430	0.0668	0.0221	0.0353	0.2062	Noise_of_short_axis_length_in_whole_cell
CCV116_A1B	0.0407		-0.364	-0.452	-2.732	0.985	0.2179	0.2029	0.0121	0.1215	Noise_of_axis_ratio_ratio
CCV112_C	0.0415		0.769	2.531	-0.621	1.032	0.1524	0.0151	0.1745	0.1138	Noise_of_distance_between_middle_point_of_neck_and_mother_center
A120_A	0.0416		-1.275	-0.708	-0.521	1.092	0.0878	0.1610	0.1924	0.1072	Total_length_of_actin_patch_link
ACV122_C	0.0424		0.699	2.670	[illegible]	[illegible]	0.1618	0.0121	0.2746	0.2475	Noise_of_number_of_bright_actin_patches
D175_A1B	0.0444		1.031	0.634	2.244	-1.077	0.1138	0.1725	0.0227	0.1082	Maximal_distance_between_nuclear_gravity_center_and_nuclear_outline
A8-1_A1B	0.0450		1.844	2.628	0.836	2.984	0.0397	0.0126	0.1411	0.0075	Total_brightness_of_actin_region_in_mother
CCV111_A1B	0.0450		-0.217	2.715	1.376	0.234	0.2402	0.0112	0.0758	0.2372	Noise_of_distance_between_bud_tip_and_mother_short_axis_extension
C116_C	0.0450		1.308	2.465	1.110	-0.690	0.0827	0.0162	0.1044	0.1632	Axis_ratio_ratio
A102_C	0.0450		1.947	2.541	2.849	0.725	0.0342	0.0146	0.0091	0.1574	Bud_actin_region_ratio_to_total_region
A7-2_C	0.0452		2.210	3.311	2.113	2.439	0.0254	0.0057	0.0288	0.0181	Size_of_actin_region_in_bud
DCV192_C	0.0455		1.711	3.102	0.972	0.624	0.0488	0.0066	0.1226	0.1742	Noise_of_average_of_nuclear_brightness_in_bud
CCV11-1_A	0.0465		0.342	1.466	1.115	-3.184	0.2210	0.0700	0.1048	0.0066	Noise_of_whole_cell_size
DCV127_A	0.0465		-0.999	-0.453	[illegible]	-2.820	0.1185	0.2029	0.2452	0.0094	Noise_of_distance_between_nuclear_brightest_point_and_cell_tip
C117_C	0.0465		2.330	1.314	1.565	3.236	0.0197	0.0824	0.0598	0.0054	Cell_outline_ratio
A9_C	0.0465		2.172	5.207	2.974	2.199	0.0276	0.0008	0.0093	0.0266	Proportion_of_actin_region_at_neck
D103_C	0.0465		-2.005	-1.259	-0.888	-2.540	0.0334	0.0895	0.1343	0.0160	Distance_between_nuclear_gravity_center_in_mother_and_mother_tip
DCV195_C	0.0465		-1.205	-1.459	-2.625	-3.047	0.0937	0.0696	0.0129	0.0070	Noise_of_maximal_intensity_of_nuclear_brightness_divided_by_average_in_bud
D203	0.0469		-1.803	-2.210	-1.210	-2.380	0.0420	0.0237	0.0929	0.0180	Nuclear_D_ratio
DCV173_A	0.0470		1.220	3.129	2.116	1.777	0.0929	0.0070	0.0288	0.0458	Noise_of_maximal_distance_between_nuclear_gravity_center_and_nuclear_outline
DCV184_A1B	0.0470		-3.320	[illegible]	-3.559	-1.767	0.0056	0.2523	0.0042	0.0463	Noise_of_nuclear_axis_ratio
DCV148_A1B	0.0471		[illegible]	1.859	2.685	0.826	0.2624	0.0387	0.0117	0.1422	Noise_of_relative_distance_of_nuclear_brightest_point_to_mother_center
D15-2_C	0.0471		3.266	4.525	4.400	1.426	0.0060	0.0013	0.0015	0.0732	Nuclear_brightness_in_bud
D15-3_C	0.0489		2.726	4.616	4.400	1.907	0.0121	0.0012	0.0015	0.0379	Nuclear_brightness_in_whole_cell
DCV188_C	0.0489		1.243	2.970	1.309	2.589	0.0903	0.0079	0.0827	0.0138	Noise_of_distance_between_nuclear_gravity_center_and_brightest_point_in_mother
C118_C	0.0495		1.736	0.502	1.378	3.054	0.0467	0.1947	0.0757	0.0068	Cell_size_ratio
D134_C	0.0496		-2.204	-3.156	-2.282	-0.936	0.0254	0.0068	0.0230	0.1280	Distance_between_two_nuclear_brightest_points_through_middle_point_of_neck
DCV116_C	0.0496		1.862	[illegible]	2.355	0.402	0.0389	0.2509	0.0193	0.2110	Noise_of_distance_between_two_nuclear_gravity_centers_through_middle_point_of_neck
